# Seasonal patterns in risk factors for *Taenia solium* transmission: a GPS tracking study of pigs and open human defecation in northern Peru

**DOI:** 10.1186/s13071-019-3614-5

**Published:** 2019-07-16

**Authors:** Ian W. Pray, Claudio Muro, Ricardo Gamboa, Percy Vilchez, Wayne Wakeland, William Pan, William E. Lambert, Hector H. Garcia, Seth E. O’Neal, for the Cysticercosis Working Group in Peru

**Affiliations:** 1 School of Public Health, Oregon Health & Science University and Portland State University, Portland, Oregon USA; 2 Center for Global Health Tumbes, Universidad Peruana Cayetano Heredia, Tumbes, Peru; 3 Systems Science Program, Portland State University, Portland, Oregon USA; 4 Global Health Institute, Duke University, Durham, North Carolina USA; 5 School of Sciences, Department of Microbiology, Universidad Peruana Cayetano Heredia, Lima, Peru

**Keywords:** *Taenia solium*, Cysticercosis, Cestodes, Pigs, GPS, Open defecation, Peru

## Abstract

**Background:**

*Taenia solium* (cysticercosis) is a parasitic cestode that is endemic in rural populations where open defecation is common and free-roaming pigs have access to human feces. The purpose of this study was to examine the roaming patterns of free-range pigs, and identify areas where *T. solium* transmission could occur *via* contact with human feces. We did this by using GPS trackers to log the movement of 108 pigs in three villages of northern Peru. Pigs were tracked for approximately six days each and tracking was repeated in the rainy and dry seasons. Maps of pig ranges were analyzed for size, distance from home, land type and contact with human defecation sites, which were assessed in a community-wide defecation survey.

**Results:**

Consistent with prior GPS studies and spatial analyses, we found that the majority of pigs remained close to home during the tracking period and had contact with human feces in their home areas: pigs spent a median of 79% (IQR: 61–90%) of their active roaming time within 50 m of their homes and a median of 60% of their contact with open defecation within 100 m of home. Extended away-from-home roaming was predominately observed during the rainy season; overall, home range areas were 61% larger during the rainy season compared to the dry season (95% CI: 41–73%). Both home range size and contact with open defecation sites showed substantial variation between villages, and contact with open defecation sites was more frequent among pigs with larger home ranges and pigs living in higher density areas of their village.

**Conclusions:**

Our study builds upon prior work showing that pigs predominately roam and have contact with human feces within 50–100 m of the home, and that *T. solium* transmission is most likely to occur in these concentrated areas of contact. This finding, therefore, supports control strategies that target treatment resources to these areas of increased transmission. Our finding of a seasonal trend in roaming ranges may be useful for control programs relying on pig interventions, and in the field of transmission modeling, which require precise estimates of pig behavior and risk.

**Electronic supplementary material:**

The online version of this article (10.1186/s13071-019-3614-5) contains supplementary material, which is available to authorized users.

## Background

Cysticercosis, caused by the pork tapeworm (*Taenia solium*), imposes a major health and economic burden on rural populations in Latin America, Africa and eastern Asia [1, 2]. Humans acquire the intestinal tapeworm infection (taeniasis) by consuming larval cysts that may be present in raw or undercooked pork. Adult tapeworms reside in the human intestine, and may expel tens of thousands of infectious eggs each day in the host’s feces [3, 4], which contaminate the environment in areas where open human defecation is common. The widespread practice of free-range pig-raising in endemic areas allows pigs to consume *T. solium* eggs in human feces and develop larval cyst infections in their muscle tissue, thus perpetuating the life-cycle.

The movement patterns of free-roaming pigs within endemic communities and their contact with potentially infectious human feces are key factors that influence transmission patterns. Prior studies have found that pigs raised in the same household or within 50 meters of a human with taeniasis have substantially higher rates of cyst infection [5–7] and antibody reactivity [8] compared to more distant pigs. This knowledge of locally acquired *T. solium* infection has led to important advancements in control in recent years. In Peru, “Ring Strategy” has led to significant disease control by offering screening and treatment for human taeniasis to people living within 100 meters of an infected pig [9].

Although the evidence for focal transmission of *T. solium* is convincing, there are significant gaps in our knowledge of transmission that have been highlighted by prior spatial studies. Namely, past studies have routinely found infected pigs living far from known tapeworm carriers [5, 6], and ring interventions have not completely eliminated the disease [9], as would be expected if transmission were purely focal. An improved understanding of *T. solium* transmission dynamics, including elucidation of these unexplained patterns of pig infection, would have a few key impacts on the prospects for *T. solium* control. First, it may lead to improved intervention strategies that more effectively target treatment resources to areas of transmission risk. Secondly, it would provide key information for the emerging field of transmission modeling. Existing models of *T. solium* transmission have been used to compare the effectiveness of available control strategies [10, 11], but have not yet had sufficient data to incorporate spatial aspects of transmission. Addressing this knowledge gap requires that we investigate the behavioral and environmental factors that produce the observed spatial patterns in transmission; chief among these are the roaming patterns of pigs and their contact with human feces present in the environment due to open defecation practices.

Having previously identified these goals, we first investigated the roaming patterns of pigs in a pilot study conducted in 2015 [12]. In that study, we used GPS trackers to map the roaming ranges and contact with human feces for 37 pigs in two small villages of northern Peru. That study helped to validate the size of 100-meter rings used in Ring Strategy, but was limited by a short tracking period (48 hours), a small sample of pigs from only two villages, and tracking during the rainy season only, all factors that could have led to biased or imprecise estimates.

In the present study, we set out to further investigate the roaming patterns of pigs in this region with the goal of improving upon the limitations of our pilot study. Specifically, this study expanded to three new villages in northern Peru, included more pigs (*n *= 108), a longer tracking period (up to six days), and tracking in both the rainy and dry seasons.

## Methods

### Selection of study villages and tracking seasons

Three villages in the northern Peruvian region of Piura participated in this study. We selected these villages (herein referred to as villages “A”, “B” and “C”) because they were generally representative of rural villages in the region, had an adequate number of households that raised free-roaming pigs, and were participating in a concurrent cysticercosis control study that provided up-to-date census information [13]. Four other villages located in the region and also participating in the over-arching study were excluded because our logistical capacity was limited to three villages, and the excluded villages had fewer households that reported raising free-roaming pigs. The period of GPS tracking referred to as “rainy-season” tracking took place in the study villages in April 2018, which corresponds to the end of the rainy season (December-April) and is characterized by intermittent rain and abundant wild fruits and foliage. “Dry-season” tracking took place in the same villages in August 2018, a period characterized by cool and dry weather with very little green foliage.

### Sample size

The sample size of pigs for this study was designed to explore differences between home-range areas by season (two-sided, α= 0.05). Our chosen sample size of 120 pigs (20 pigs per village per season) corresponded to an 80% power to detect a 35% difference in median home range by season in the full sample and 54% seasonal difference within each village stratum. Calculations were based on mean and variance results from our pilot study in this region [12].

### Selection of pigs

All households in participating villages were approached for inclusion in the study and were eligible if they reported raising free-roaming pigs. At consenting households, pigs were eligible for GPS tracking if they were not regularly tied or enclosed in a corral, were at least two months old, were not pregnant or sick, and were not planned for slaughter in the next seven days. We attempted to enroll one pig from each consenting household. If multiple pigs could be captured from one household, we enrolled the pig that fulfilled an age-stratified sampling scheme. For dry season tracking, we enrolled the same pigs that participated in the rainy season when possible. If this pig had been sold or slaughtered, we selected a pig from the same household with preference towards pigs that were the same age as the previously tracked pig.

### GPS tracking of pigs

**Fig. 1:**
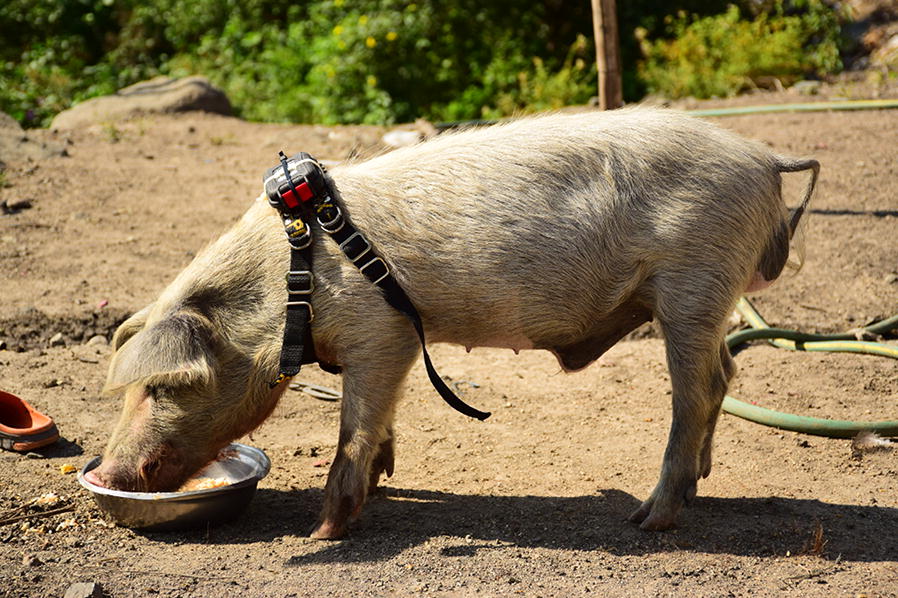
GPS devices placed in waterproof cases and secured to harnesses for tracking

The GPS loggers we used for this study (“i-GotU GT-120”; MobileAction Technology, New Taipei City, Taiwan) were programmed to record the GPS coordinates of a pig’s location every 60 s. In order to last the planned 6-day roaming period at this logging frequency, we replaced the original 230 mAh batteries with 3.7 V, 2000 mAh lithium-ion batteries (AdaFruit, New York, NY, USA) in all devices used. After each pig was captured, the modified GPS logger was placed in a waterproof case (HPRC 1100; Plaber, Vicenza, Italy) and secured to the nape of the pig using a custom harness made of nylon webbing (Fig. 1). All study pigs from each village were tracked over the same 6-day period. During this period, study staff returned to each enrolled household daily to check on pigs and adjust harnesses if necessary. At the end of the 6-day period, the GPS devices were removed and the spatial data were downloaded for analysis.

### Household defecation survey

In addition to tracking pigs, we conducted household surveys to assess human defecation practices in the study villages. For this, we visited all households during the rainy season and asked available adult residents whether their family owned a latrine/indoor bathroom or members of their family practiced open outdoor defecation. If an outdoor area was indicated, we searched for evidence of recent defecation (e.g. feces or soiled paper) and used a handheld GPS receiver (GeoExplorer II; Trimble, Sunnyvale, CA, USA) to record a GPS point at that location. For both latrines and outdoor defecation areas, household respondents were asked to rate their family’s frequency of use between “never”, “sometimes” or “always”. Finally, study teams logged the locations of roads, paths and streams in the community and inspected each for evidence of open human defecation. Study personnel were assisted in this effort by local community leaders who guided teams to known communal defecation sites in each village.

### Mapping and statistical analysis

All data were analyzed using R v.3.2 (R Foundation for Statistical Computing, http://www.r-project.org), QGIS v.2.18 (Open Source Geospatial Foundation Project, http://qgis.osgeo.org) and Stata v.13.1 (StataCorp, College Station, TX, USA). For spatial analyses, all spatial layers were projected with a Universal Transverse Mercator Zone 17S projection. Because obstruction of the satellite signal occurred intermittently during pig tracking, it was necessary to remove outlying points in post-processing. To do this, we removed points that were delayed > 10 s (suggesting signal obstruction), points for which the detected speed was greater than 3 m/s and points with less than a 20° angle between the prior and succeeding GPS locations, features unlikely to be produced by natural pig movement. On average, we removed 3.1% of the total points logged for each pig due to suspected error. Additionally, in order to avoid bias due to the stress of the chase and capture of pigs, we removed the first hour and final 15 min of tracking time, as well as points that were recorded before, during, and after any necessary harness adjustments.

In order to create maps that represented the active foraging time for pigs, when they are most likely to consume human feces, we further restricted the GPS points included in the analysis by two factors. First, we excluded points taken between 22:00 and 4:00 h, a time in which most range maps showed inactivity for pigs, and secondly, we excluded points for which the GPS coordinates did not change from the preceding point, suggesting inactivity. We validated this method of selecting for active foraging time by directly observing the behaviors of a subset of study pigs (*n *= 9) in the field. For these pigs, which were each observed for 12 daytime hours, we found that removing repeat points successfully eliminated non-foraging rest-time with a sensitivity of 38% and specificity of 96%. Overall, these additional filters reduced the total number of GPS points used for each pig from an average of 7727 total points to 4569 active points, a 37% reduction.

**Fig. 2:**
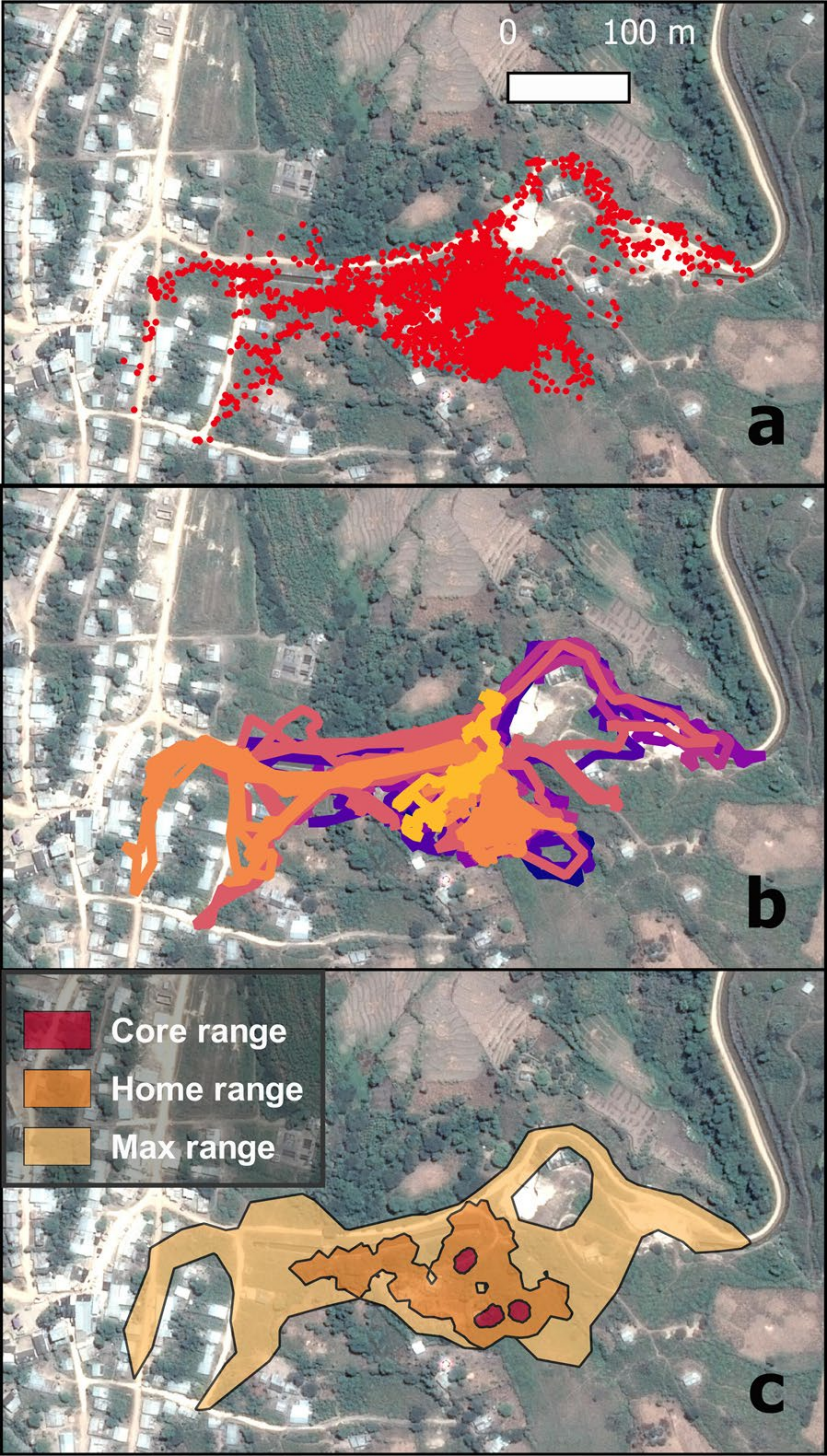
**a** Map of raw GPS points from a single pig (Village B). **b** Line map of the same pig’s roaming pattern with each color representing a unique day of movement. **c** Final LoCoH map of the same pig’s range with colors representing core (50%), home (90%) and maximum (100%) range levels. Satellite images from Google Satellite Hybrid extension for QGIS. Last update April 05, 2017

After obtaining final datasets for each pig, we analyzed roaming ranges using the “LoCoH” (localized convex hulls) Homerange Analysis Algorithm for R [14, 15]. A detailed description of the LoCoH algorithm can be found elsewhere [16]. Briefly, we used the *a*-nearest-neighbors LoCoH method (*a* for adaptive), which is a non-parametric mapping algorithm that creates convex polygon hulls around each GPS point based on a flexible number of nearest-neighbor points. The *a-*method uses fewer nearest-neighbor points to constructs hulls in less dense areas of the range, thus avoiding the problem of large polygons forming in sparsely occupied areas. We found that the algorithm produced optimal roaming areas when the “*auto-a*” function required a minimum of 95% of points to form polygons with 30 nearest neighbors. The output of the LoCoH algorithm produced maps of each pig’s range that identified three areas based on specified isopleth cut-off values. As suggested by the algorithm developers [16], the “core range” represents the densest 50% of a pig’s range, the “home range” is the densest 90% and the “maximum range” is the area that contained 100% of the convex hulls (Fig. 2).

In order to analyze pig roaming ranges with respect to land features and open defecation areas, we created detailed vector maps for each study village. For this, Google Earth satellite images (Google Satellite Hybrid extension for QGIS; last update April 05, 2017; map location: 4°38′12.84″S, 79°59′29.87″W) were overlaid with manually logged household and road layers to categorize village land into one of four mutually exclusive land types: peri-domestic, roads/paths, farmland and vegetation. Peri-domestic areas were formed by generating 20-m buffers around household coordinates and merging the areas surrounding contiguous households and common areas (e.g. school, recreational fields, etc), roads and paths were manually logged in the field and enhanced with a 4-m buffer in post-processing, farmland was assigned in post-processing by digitizing visible fence-lines that contained discernible rows of crops, and all remaining areas not fitting these categories were classified as vegetated (these remaining areas were composed of undeveloped land with sparse tree cover, bushes and streams).

We processed LoCoH maps with respect to these base layers in order to extract a variety of roaming outcomes. These included the total area of core, home and maximum LoCoH ranges, the proportion of tracking time spent in each land type, the number of human defecation points within each level of a pig’s range (core, home and maximum ranges) and their corresponding land types, and distance of each GPS point to the pig’s household, which was used to determine the proportion of time spent within 50, 100, 150 and 200 m of home.

Roaming outcomes were first analyzed descriptively and were then analyzed for associations with pig-, household- and village-level predictors. These predictors included pig age (in months), sex, household herd size, household density (number of neighboring households within 100 m), village of residence and tracking season. These predictors were used to create a variety of multivariable models for pig roaming: ordinary least squares regression models for the log-area of core, home and maximum ranges; negative binomial models for the number defecation points inside pigs’ home and maximum ranges; and a logistic regression model for the presence of at least one open defecation site within a pigs’ core ranges. Predictors and interactions were retained in either model if they were significant (*P *< 0.05) when added in stepwise procedure. Because of similarities in the results of our models for core, home and maximum ranges, only the results of the two home-range analyses are presented here, but all models and corresponding coefficients are provided in Additional file 1: Tables S2 and S3.

## Results

### Village and household characteristics

All three study villages are rural communities where small-holder farming is the primary economic activity and raising free-roaming pigs is common practice. Between 53 and 70% of households reported raising pigs and only 5–29% of those pig-owners reported always corralling their pigs (Table 1). Despite similar population sizes (range: 83–95 households), the three study villages had important differences. Village A was larger, flatter and less densely housed than the other two villages, while Villages B and C were smaller and built on steep sloping terrain. Village B was the smallest and densest village characterized by fewer latrines, a higher rate of open defecation and significantly more open defecation sites.

**Table 1:** Characteristics of study villages and defecation survey

	Village A	Village B	Village C
Human population	279	250	372
Households	95	83	83
Household density^a^	6.9	26.1	11.2
Area (km^2^)	1.93	0.45	0.58
Participated	77/95 (81%)	70/83 (84%)	79/83 (95%)
Latrine prevalence	74/77 (96%)	46/70 (66%)	75/79 (95%)
Open defecation^b^	13/77 (17%)	32/70 (46%)	25/79 (32%)
Total no. of defecation sites	30 (20%)	79 (52%)	42 (28%)
No. of pig owners	41/77 (53%)	45/70 (64%)	55/79 (70%)
Corral prevalence	31/41 (76%)	17/45 (38%)	18/55 (33%)
Actual corral use^c^	12/41 (29%)	6/45 (13%)	3/55 (5%)

^a^Mean no. of households within 100 m

^b^Some houses with latrines also reported open defecation

^c^Corral in “good” condition and owner reports that it is used “always”

### Pig population

We enrolled a total of 114 pigs for GPS tracking between the two seasons. Six pigs were excluded from the analysis because of a combination of device failure (*n *= 3), lost devices (*n *= 2) and an owner’s decision to corral the pig (*n *= 1). This led to a final sample of 108 pigs tracked: 53 in the rainy season and 55 in the dry season. Of the 53 rainy season pigs, we were able to repeat dry season tracking for 15 pigs (28%) and track a pig from the same household for 37 pigs (70%). There were no significant differences in the sex, age or village distribution of pigs between the rainy and dry seasons (see Additional file 1: Table S1).

Pigs included in the analysis were tracked for an average of 5.4 days (range: 2.2 to 6.6 days). The targeted 6-day tracking period was incomplete for 21 (19%) of the 108 pigs analyzed. Reasons for incomplete tracking included premature battery death or device failure (*n *= 16), owner’s decision to withdraw (*n *= 4) and pig death (*n *= 1, unrelated to study).

### Household distance and defecation contact

**Fig. 3:**
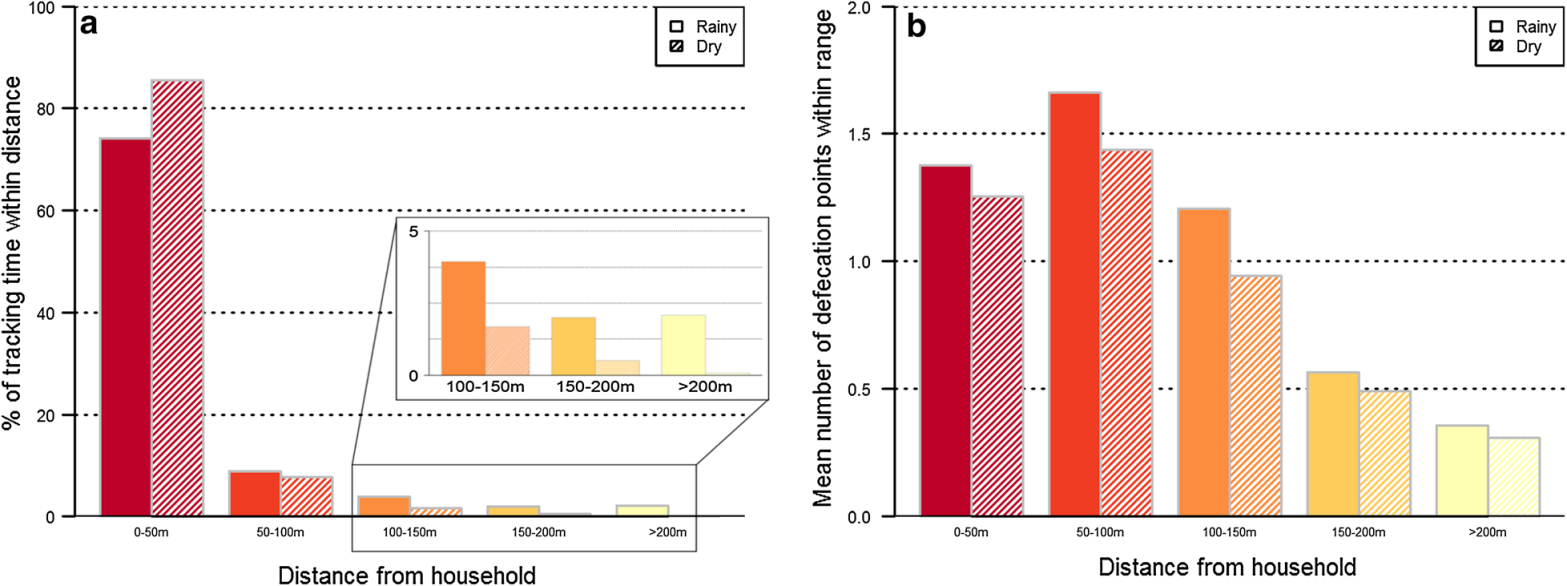
**a** The median proportion of active time pigs spent at increasing distances from their households in rainy (*n *= 53) and dry (*n *= 55) seasons. **b** The mean number of defecation points within the maximum LoCoH range of pigs at increasing distances from their households in rainy and dry seasons

We first analyzed the amount of time pigs spent at increasing distances from their homes. In both tracking seasons, pigs spent the majority of their-active time within 50 m of their homes (medians: 74% in rainy, 85% in dry, Wilcoxon rank-sum test: *Z *= − 1.91, *P *= 0.056; Fig. 3a). The proportion of active roaming time spent at increasing distances decreased substantially outside of 50 m in both seasons. The median proportions of active time spent in rainy and dry seasons were respectively 8.8% and 7.8% at 50–100 m, 3.9% and 1.7% at 100–150 m, 2.0% and 0.5% at 150–200 m, and 2.1% and 0.7% at > 200 m.

Despite spending the majority of total time very close to households, distances at which contact with human defecation sites occurred followed a different pattern and did not differ significantly between seasons (Fig. 3b). In both seasons, the majority of contact between pig ranges and defecation sites occurred between 50 and 100 m of the household (mean number of defecation sites contacted within 50–100 m of home: 1.66 in rainy season, 1.43 in dry season, t-test: *t*_(106)_ = 0.55, *P *= 0.58). The number of defecation contacts decreased at increasing distances from the household, but was disproportionately large at long distances compared to the total time pigs spent at those distances.

### Roaming range areas

**Fig. 4:**
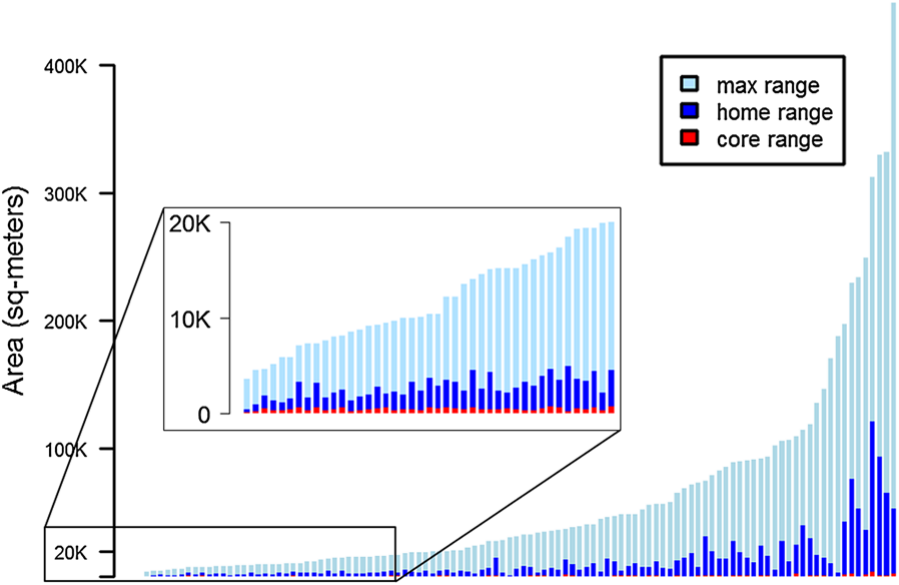
Areas of LoCoH core, home and maximum ranges for all 108 pigs tracked

The areas of core, home and maximum ranges are shown for all pigs in Fig. 4. Range sizes were distributed exponentially, with the majority of pigs having maximum range areas of less than 30,000 m^2^ and home range areas less than 5000 m^2^. However, a subset of pigs had ample roaming ranges that revealed regular extended trips to distant areas. In these extreme cases, pigs ventured 1–3 km from their homes, and spent nights away without returning home. For these pigs, maximum ranges reached 500,000 m^2^ with home range areas up to 120,000 m^2^.

**Fig. 5:**
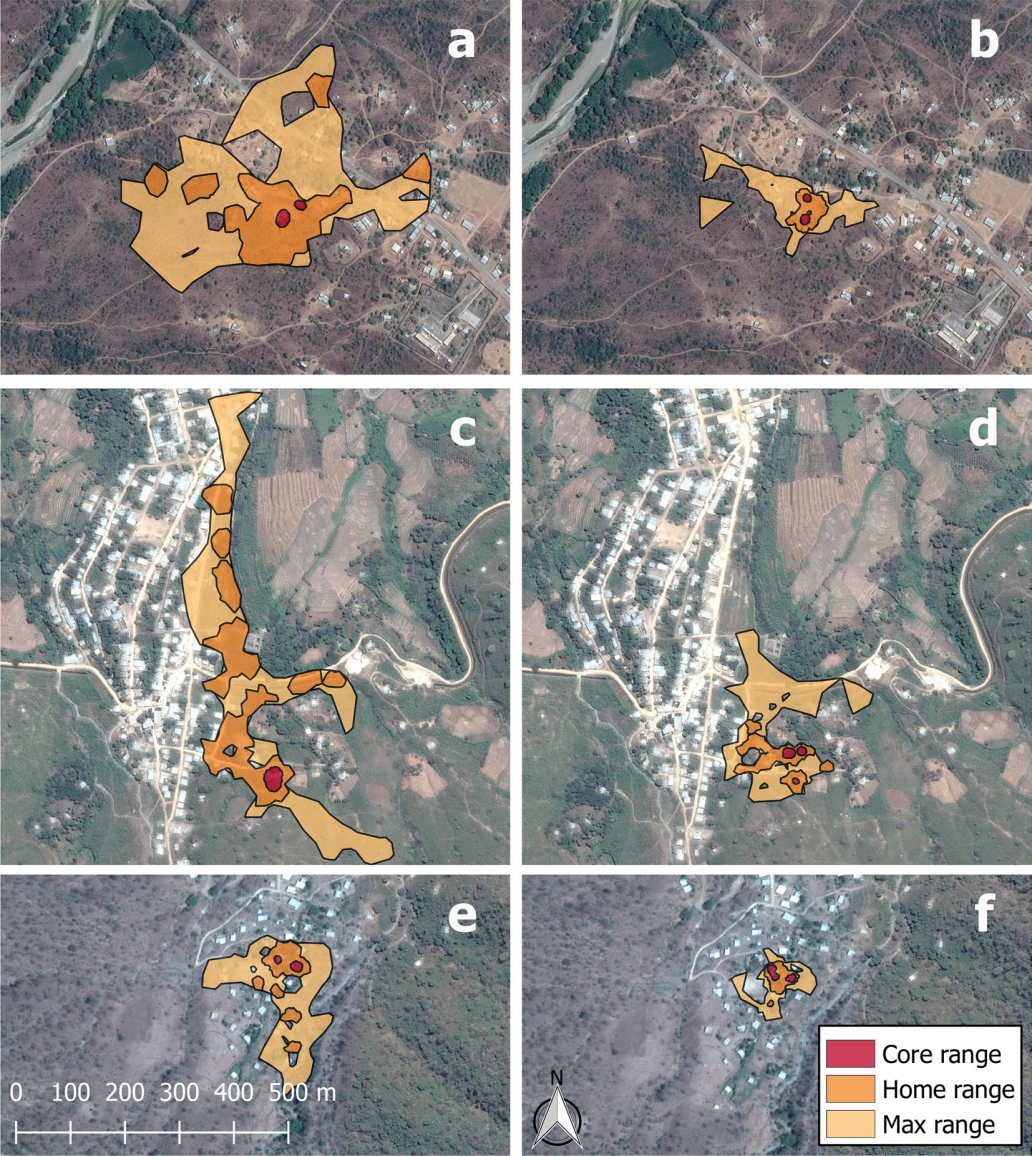
LoCoH home range maps of 6 representative pigs from 3 study villages. **a** Village A, rainy season. **b** Village A, dry season, **c** Village B, rainy season. **d** Village B, dry season. **e** Village C, rainy season. **f** Village C, dry season. Adjacent maps are from pigs of the same household in the rainy and dry seasons. LoCoH range levels represent densest 50% (core), 90% (home) and 100% (maximum) of roaming area. Satellite images from Google Satellite Hybrid extension for QGIS. Last update April 05, 2017

In multivariable regression models, village of residence and season were the only variables significantly associated with log-transformed LoCoH areas. Age, household herd size, and household density all had significant bivariate associations, but became non-significant after adjustment for village and season, and pig sex was not significant in any model (Table 2). Across all villages, home ranges were 61% (95% CI: 47-72%) smaller in the dry season, compared to the rainy season, and there was significant variation in home range areas by village. Figure 5 shows representative maps of 3 pigs tracked in both seasons.

**Table 2:** Regression coefficients for home range area and defecation sites in home range. Bivariate and multivariate linear regression models for log-area of home range, and negative binomial models for the number of open defecation sites within home ranges

	Home range area, *e*^β^ coefficients (95% CI)		Defecation sites in home range, incidence rate ratio (95% CI)	
	Bivariate	Multivariate	Bivariate	Multivariate
Village				
Village A	Ref.	Ref.	Ref.	Ref.
Village B	0.48 (0.30–0.76)**	^b^0.47 (0.31, 0.70)**	7.06 (3.83–13.01)**	^b^7.94 (4.28–14.7)**
Village C	0.24 (0.15–0.39)**	^b^0.23 (0.16, 0.35)**	1.25 (0.63–2.49)	^b^1.25 (0.57–2.70)
Season				
Rainy	Ref.	Ref.	Ref.	–
Dry	0.40 (0.27–0.59)**	^b^0.39 (0.28,0.53)**	0.69 (0.39–1.21)	–
Household density^a^				
≤ 25	0.95 (0.92–0.97)**	–	1.03 (1.00–1.07)	1.07 (1.04–1.10)**
> 25	1.05 (1.02–1.09)**	–	1.03 (0.99–1.08)	0.95 (0.93–0.98)**
Herd size (per additional pig)	1.06 (1.02–1.10)**	–	0.97 (0.91–1.03)	–
Pig sex				
Female	Ref.	–	Ref.	Ref.
Male	0.78 (0.51–1.19)	–	0.94 (0.54–1.66)	^b^1.45 (1.01–2.08)*
Pig age (per month)	1.04 (1.0–1.08)*	–	0.98 (0.93–1.03)	–
Log-area of home range	–	–	1.50 (1.13–2.0)**	1.76 (1.43–2.16)**

*P < 0.05, **P < 0.01

^a^Number of households within 100 m radius, linear spline at 25 households/100 m

^b^Significant statistical interactions (by village) not shown (see Additional file 1, Tables S2 and S3 for full model associations)

**Fig. 6:**
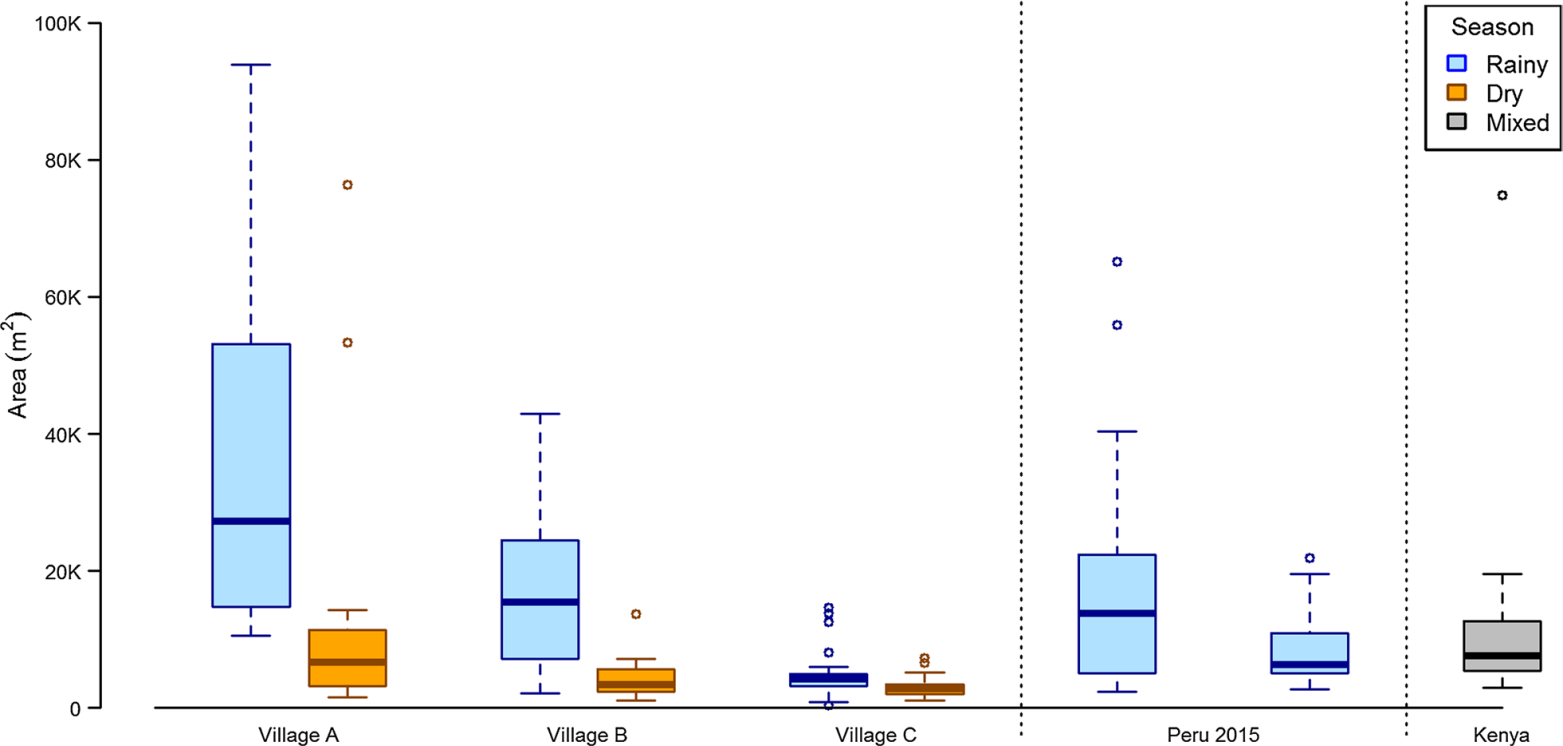
Box plots of home range areas by season and village show significant reduction in home ranges by season and between villages. Additional boxes show the home ranges extracted from pilot study in Peru [12], *n *= 37 pigs in rainy season and GPS tracking of 10 pigs in Kenya [18] from a mix of rainy and dry season tracking

The degree of reduction observed between the rainy and dry seasons was significantly different in between villages (likelihood ratio test: *χ*^2^= 9.46, *df *= 2, *P *= 0.009 for village × season interaction). Villages A and B had significant reductions of 76 and 71%, respectively, from the rainy to dry seasons, and Village C, the village with the smallest home ranges overall, had a non-significant 30% reduction in home range area. Home range areas by season and village are shown in Fig. 6, and full tables of all regression outputs, including regression models for core and maximum ranges can be found in Additional file 1: Table S2.

### Contact with defecation sites

Overall, 56% of pigs had at least one defecation site in their home range and 85% had at least one defecation site in their maximum range. The rate of contact with defecation sites was not significantly different between the rainy and dry seasons (mean of 2.1 *vs* 1.5 defecation sites in home ranges during the rainy *vs* dry seasons, t-test: *t*_(106)_ = 1.34, *P *= 0.18), but did vary significantly between villages. Pigs from Village B had an average of 4.0 defecation areas in their home ranges, compared to averages of 0.6 and 0.7 in Villages A and C, respectively (ANOVA, *F*_(2, 107)_ = 33.4, *P *< 0.0001). Of the three study villages, Village B was the village with the smallest land area, the highest density of households and by far the most defecation sites found overall.

In a negative binomial model of contact with defecation sites (Table 2), residence in Village B, male sex, increased housing density up to 25 households/100-m radius, and increased home-range area were significantly associated with the rate of contact with defecation sites. Tracking season, pig age and herd size were not significantly associated with defecation contact (see Additional file 1: Table S2).

### Pig roaming and land type

We also analyzed the amount of active time pigs spent roaming in different land types. Overall, pigs spent the majority of active roaming in the peri-domestic habitat, while proportionally less time was spent in vegetation and roads/paths, and very little time was spent in farmland. Season, village, household density and home-range size were all significantly associated with roaming land type (Table 3). Pigs spent significantly more time in peri-domestic areas during the dry season (64 *vs* 55%, t-test: *t*_(106)_= − 2.05, *P *= 0.04), and were more likely to spend time in peri-domestic areas if they had smaller home ranges (linear regression *β *= − 0.088 for log-increase in home-range area, *F*_(1,106)_= 20.3, *P* < 0.0001), or lived in higher-density areas of the village (66% for > 10 households within 100 m *vs* 54% otherwise, t-test, *t*_(106)_= − 2.73, *P *= 0.008). Contact with open defecation sites occurred most frequently in peri-domestic and vegetated zones, less frequently along roads/paths, and was not observed in farmland (mean defecation sites in range= 2.0, 1.9, 0.9 and 0, respectively).

**Table 3:** Pig roaming land type by selected covariates. Mean percentage (95% CI) of active roaming time spent in given land type. Farmland not shown due to infrequent roaming; other pig variables not shown (pig sex, age, and household herd size) were not significantly associated with any roaming land type

	Peri-domestic	Vegetation	Roads/paths
Season^a^			
Rainy	54.8 (48.7–60.9)*	26.7 (20.8–32.7)	17.3 (12.8–21.8)
Dry	64.2 (57.3–71.1)*	20.1 (13.6–26.6)	15.4 (11.3–19.5)
Village^b^			
Village A	64.9 (57.2–72.7)**	26.2 (18.2–34.4)	8.5 (6.1–10.9)**
Village B	46.1 (37.6–54.5)**	26.1 (18.0–34.3)	26.0 (20.0–32.1)**
Village C	67.9 (61.5–74.3)**	18.8 (11.6–25.9)	13.3 (8.9–17.6)**
Home-range size^a^			
< 3000 m^2^	73.8 (67.7–79.8)**	9.6 (0.6–13.4)**	16.5 (10.1–23.0)
> 3000 m^2^	54.6 (49.1–60.2)**	28.2 (22.8–33.6)**	16.2 (12.8–19.7)
Household density^a,c^			
≤ 10	53.8 (47.0–60.5)**	29.1 (22.9–35.2)**	16.7 (12.1–21.3)
> 10	66.1 (60.2–72.1)**	16.9 (11.0–22.9)**	15.9 (12.0–19.8)
No. of open defecation sites in range (mean ± SD)	1.99 ± 2.3	1.94 ± 2.5	0.87 ± 1.2

*P < 0.05, **P < 0.01

^a^Two-sample t-test used to derive *P*-values and 95% confidence intervals

^b^One-way analysis of variance (ANOVA) used to derive *P*-value and 95% confidence intervals

^c^Number of households within 100 m radius

## Discussion

The purpose of this study was to examine the roaming patterns of pigs in northern Peru, and to identify areas within their ranges where *T. solium* transmission could occur *via* contact with human feces. We found that pigs spent the majority of their active roaming time within 50 m of their household. This home-centered range was concentrated in the peri-domestic habitat and predominated across both seasons and all villages (median: 79% of active time within 50 m). Most of the areas of overlap between defecation sites and pig roaming ranges were found in this 50-m zone or the wider 100-m radius surrounding pig homes, suggesting that the majority of *T. solium* transmission risk is concentrated in these areas proximal to pigs’ households.

These findings are generally consistent with our knowledge of limited pig roaming and focal *T. solium* transmission in this region. Prior spatial analyses of tapeworm carriers and infected pigs have found that pigs living with 50 m of a tapeworm are at significantly elevated risk of cyst infection [5, 6] and our pilot GPS analysis of pig roaming in this region found that pigs spent 70% of their roaming time and 93% of their interactions with defecation sites within 50 m of their homes [12]. Taken together, these studies provide consistent and convincing evidence that the *T. solium* transmission in this region occurs in close proximity to the home, areas where pig roaming and human defecation are concentrated, and that interventions targeting treatment resources to these hotspots of transmission are likely to be successful.

Although most pigs had limited roaming ranges and close contact with human feces near their home, many pigs spent at least some fraction of time foraging in more distant areas, and a subset of pigs had ample roaming ranges that revealed regular extended trips to distant areas. In these extreme cases, pigs ventured 1–3 km from their homes and spent nights away without returning home. These long-distance roamers are an important sub-group to consider in the context of control interventions, as they had higher rates of contact with open defecation areas and, due to extended time away from home, may not be included in treatment, vaccination or serological monitoring programs.

Another key finding in this study was the importance of season as a determinant of the area and distance pigs covered during roaming. Nearly all occurrences of extended roaming were observed during the rainy season, and rainy season home ranges were 61% larger than their dry season counterparts. Compared to the dry season, pigs in the rainy season also spent less time foraging in peri-domestic zones. This seasonal pattern is likely due to the increased availability of wilds fruits, vegetation and natural streams during the rainy season. Pig owners frequently reported to us that their pigs roamed longer and further during the rainy summer months in search of wild fruits to eat and streams to bathe in, and spent the dry winter months resting and grazing on domestic food sources. While we did not collect information about the provision of pig feed by owners, we have observed that purchased feed in this region is rare due to its cost, suggesting that the availability of natural food sources and not pig feeding patterns is the most likely explanation for seasonal differences in roaming ranges. This seasonal pattern is consistent with a non-spatial study of pig behavior conducted in Mexico, which found that pigs spent more time feeding and walking during the rainy season, and more time resting and consuming feces during the dry season [17]. Despite our finding of seasonality in roaming range areas, we did not detect any significant difference in contact with human feces between seasons, and therefore were not able to corroborate evidence of a seasonal pattern in *T. solium* transmission.

Apart from season, the most important determinant of the size of a pig’s roaming area and its contact with defecation areas was its village of residence. Roaming areas in Village A were considerably larger than those observed in Villages B or C (median home ranges: 12,570; 5697; and 3270 m^2^, respectively), yet contact with defecation sites was more frequent in Village B (mean of 4.0 defecation sites in range *vs* 0.6 and 0.7 in Villages B and C, respectively). These differences highlight the importance of village-specific characteristics that may lead to heterogeneous transmission patterns between villages. For example, Village A is relatively flat with large and dispersed homesteads (6.9 households/100 m) and a low rate of open defecation (97% of households had latrines), while Village B is a densely populated peri-urban settlement (26.1 households/100 m) with a high rate of open defecation (only 66% of households owned latrines). Given that pig roaming patterns and contact with open defecation areas varied considerably between these villages, it is likely that spatial patterns of transmission and the degree of clustering in *T. solium* transmission differ as well. Control programmes should consider the impact of these between-village heterogeneities when planning interventions. For example, the decision to select a mass or focal intervention may differ depending on the degree of clustered transmission likely to be present. Knowledge of the local patterns in pig roaming, open defecation and housing density may help to tailor intervention strategies local conditions.

This study had a few important strengths compared to prior research in this field. First, repeated tracking periods allowed us to investigate seasonal differences in roaming patterns. This aspect of pig roaming was not addressed in our prior analysis, and was not robustly evaluated in two other studies relating pig roaming to *T. solium* transmission risk: a GPS study in Kenya that tracked five pigs per season [18] and a non-spatial study of pig behavior in Mexico [17]. Our study tracked over 50 pigs per season across three villages, the most robust effort to date to study pig behavior as it relates to *T. solium* transmission. Secondly, our application of a six-day tracking period (compared to two days in our prior study) and our selection of active roaming time were key improvements that reduced the impact of chance daily variations in roaming and the introduction of bias from periods of rest that would not contribute to transmission risk.

Despite these strengths, our study has a few important limitations. Due to the logistical challenges of mapping defecation sites in the communities, defecation mapping was only applied in the rainy season, and defecation sites were assumed to remain constant in the dry season. Although we are not aware of evidence from literature or local experts that open defecation practices vary by season, this remains a possibility and could have affected estimates of contact with defecation in the dry season. Secondly, while we applied multiple measures to eliminate erroneous GPS points caused by signal disruption, some degree of imprecision in GPS points was inevitable. GPS imprecision likely introduced random error into our classification of pigs’ land usage and reduced the accuracy of our algorithm to select periods of active roaming. Finally, roaming patterns and patterns of contact with human feces likely differ between endemic regions, and results obtained from these three villages may not be generalizable to other areas. In fact, the substantial differences in roaming patterns and defecation practices that were observed between the three culturally and geographically related villages included in this study suggest that even more extreme differences would be expected in other regions and continents where *T. solium* is endemic. Therefore, it will be important to replicate this work in other endemic areas in order to compare the spatial patterns of *T. solium* transmission and the generalizability of our findings to these regions. With that said, our findings from Peru are comparable to the limited prior work on this topic from Kenya and Mexico [17, 18] (see Fig. 6), and spatial analyses from Latin America and sub-Saharan Africa that have detected clustered patterns of *T. solium* prevalence [5–8, 19, 20].

## Conclusions

We found that the majority of pig roaming and contact with human defecation sites occurred in close proximity to pigs’ homes: roaming was concentrated within 50 m and contact with human defecation within 100 m of pigs’ households. When considered alongside prior GPS tracking studies and spatial analyses in this region, this study provides strong evidence that *T. solium* transmission is most likely to occur in close proximity to the home and supports control strategies that target treatment resources to these high-risk areas. When longer-range pig roaming occurred, it occurred more frequently in the rainy season and varied between villages. Therefore, while we did not find evidence that contact with feces varied by seasonal or village-specific factors, we recommend that future control strategies consider these factors when planning interventions such as pig treatment or vaccination as they could impact availability of pigs for participation. The information provided here may also be useful for *T. solium* transmission models, which require precise estimates for behavioral factors that influence transmission patterns, such as pig roaming and open human defecation. Pig roaming and open human defecation are key features that cause clustered patterns of *T. solium* transmission, and modelers should account for this clustering, along with possible seasonal and village-specific differences in transmission patterns when considering the structure and parameterization of future models. Ultimately, data from this study may fill an important gap in behavioral data needed for the development of accurate and validated *T. solium* transmission models. Advancements of *T. solium* modeling, including improved biological and behavioral data, is a need that has been highlighted by the World Health Organization as a priority for achieving control and elimination milestones [21].

## Additional file

**Additional file 1: Table S1.** Characteristics of tracked pigs. **Table S2.** Regression coefficients for the log-area of maximum, home and core roaming ranges. **Table S3.** Regression coefficients for the number of open defecation sites within pig range areas (maximum, home and core ranges).

## Data Availability

The data collected for this study are available from the corresponding author upon request.

## Abbreviations

GPS: global positioning system
IQR: interquartile range
CI: confidence interval
LoCoH: localized convex hulls
SD: standard deviation
ANOVA: analysis of variance
