# Supplementary material for: Seasonal patterns in risk factors for Taenia solium transmission: a GPS tracking study of pigs and open human defecation in northern Peru
Source: Parasit Vectors. 2019 Jul 16;12:352. doi: 10.1186/s13071-019-3614-5 (PMC6636017; doi:10.1186/s13071-019-3614-5)
Supplement: Supplementary file 1 — Additional file 1: Table S1. Characteristics of tracked pigs. Table S2. Regression coefficients for the log-area of maximum, home and core roaming ranges. Table S3. Regression coefficients for the number of open defecation sites within pig range areas (maximum, home and core ranges). [file 13071_2019_3614_MOESM1_ESM.docx]

**Additional file 1**

**Table S1. Characteristics of tracked pigs**

|  | **Village A** | | **Village B** | | **Village C** | |
| --- | --- | --- | --- | --- | --- | --- |
| **Characteristics** | **Rainy** | **Dry** | **Rainy** | **Dry** | **Rainy** | **Dry** |
| Total pigs tracked | 14 | 16 | 18 | 19 | 21 | 20 |
| Sex (male, %) | 6 (43%) | 3 (19%) | 4 (22%) | 9 (47%) | 13 (62%) | 15 (75%) |
| Age, in months (mean, sd) | 13 (6.0) | 12 (5.9) | 9 (6.9) | 9 (5.6) | 7 (4.4) | 8 (4.2) |
| Herd size (mean, sd) | 11 (7.3) | 10 (6.4) | 5 (3.3) | 7 (4.6) | 6 (3.8) | 6 (4.2) |

**Table S2. Regression coefficients for the log-area of maximum, home, and core roaming ranges.** Ordinary least squares regression models (bivariate and multivariate).

|  | **Bivariate**  **(*e*^β^ coefficients (95% CI))** | | | **Multivariate**  **(*e*^β^ coefficients (95% CI))** | | |
| --- | --- | --- | --- | --- | --- | --- |
|  | **Max. range** | **Home range** | **Core range** | **Max. range** | **Home range**^a^ | **Core range** |
| **Village** |  |  |  |  |  |  |
| Village A | Ref. | Ref. | Ref. | Ref. | Ref. | Ref. |
| Village B | 0.50**  (0.32, 0.77) | 0.48**  (0.30, 0.76) | 0.53**  (0.41, 0.69) | 0.49**  (0.33, 0.74) | ***Rainy*:** 0.43**  (0.24, 0.75)  ***Dry*:** 0.51**  (0.07, 0.22) | 0.64**  (0.50, 0.82) |
| Village C | 0.19**  (0.12, 0.29) | 0.24**  (0.15, 0.39) | 0.64**  (0.49, 0.84) | 0.19**  (0.12, 0.28) | ***Rainy*:** 0.14**  (0.08, 0.24)  ***Dry*:** 0.10**  (0.05, 0.17) | 0.52**  (0.41, 0.67) |
| **Season** |  |  |  |  |  |  |
| Rainy | Ref. | Ref. | Ref. | Ref. | Ref. | Ref. |
| Dry | 0.40**  (0.27, 0.59) | 0.40**  (0.27, 0.59) | 0.70**  (0.57, 0.88) | 0.53**  (0.38, 0.73) | ***Village A*:** 0.24**  (0.14, 0.44)  ***Village B*:** (0.29**  (0.17, 0.49)  ***Village C:*** 0.70  (0.43, 1.16) | 0.69**  (0.57, 0.84) |
| **Household density^b^** |  |  |  |  |  |  |
| ≤25 | 0.94**  (0.92, 0.96) | 0.95**  (0.92, 0.97) | 0.98*  (0.97, 0.99) | - | - | - |
| >25 | 1.06**  (1.03, 1.09) | 1.05**  (1.02, 1.09) | 1.00  (0.99, 1.02) | - | - | - |
| **Herd size (per pig)** | 1.07**  (1.03, 1.11) | 1.06**  (1.02, 1.10) | 1.01  (0.99, 1.04) | - | - | - |
| **Pig sex** |  |  |  |  |  |  |
| Female | Ref. | Ref. | Ref. | - | - | - |
| Male | 0.70  (0.45, 1.07) | 0.78  (0.51, 1.19) | 0.88  (0.70, 1.10) | - | - | - |
| **Pig age (per month)** | 1.06**  (1.02, 1.10) | 1.04*  (1.0, 1.08) | 1.02*  (1.0, 1.04) | - | - | - |

p-value: **<0.01, *<0.05

^a^Significant statistical interactions (village*season)

^b^Number of households within 100m radius, linear spline at 25 households/100m

**Table S3. Regression coefficients for the *number of open defecation sites* within pig range areas (maximum, home, and core ranges).** Negative binomial models for the number of defecation sites within maximum and home ranges (rate ratios [RR] with 95% confidence intervals [CI] displayed), and logistic regression model for the presence of ≥ 1 defecation site within the core range (odds ratios [OR] with 95% CI displayed).

|  | **Bivariate** | | | **Multivariate** | | |
| --- | --- | --- | --- | --- | --- | --- |
|  | **Max. range**  **(RR, 95% CI)** | **Home range**  **(RR, 95% CI)** | **Core range**  **(OR, 95% CI)** | **Max. range**  **(RR, 95% CI)** | **Home range^a^**  **(RR, 95% CI)** | **Core range**  **(OR, 95% CI)** |
| **Village** |  |  |  |  |  |  |
| Village A | Ref. | Ref. | Ref. | Ref. | Ref. | Ref. |
| Village B | 5.18** (3.53,7.60) | 7.06**  (3.83, 13.01) | 6.72*  (1.37, 33.0) | 5.3**  (3.61,7.78) | ***Female*:** 16.3**  (6.25, 42.3)  ***Male*:** 3.84**  (1.77, 8.39) | 21.8** (2.74, 173) |
| Village C | 1.02  (0.67, 1.56) | 1.25  (0.63, 2.49) | 1.11  (0.17, 0.30) | 1.48 (0.91, 2.41) | ***Female*:** 2.45  (0.76, 7.9)  ***Male*:** 0.63  (0.25, 1.57) | 4.59 (0.43,48.5) |
| **Home range size**  **(log-area, m^2^)** | 1.47**  (1.17, 1.84) | 1.50**  (1.13, 2.0) | 2.45*  (1.03, 5.82) | 1.87**  (1.59, 2.21) | 1.69**  (1.39, 2.07) | 5.38*  (1.50, 19.2) |
| **Household density^b^** |  |  |  |  |  |  |
| ≤25 | 1.02  (0.99, 1.05) | 1.03  (0.99, 1.07) | 1.04  (0.97, 1.12) | 1.06**  (1.05, 1.08) | 1.08**  (1.05, 1.11) | - |
| >25 | 1.04*  (1.0, 1.08) | 1.03  (0.99, 1.08) | 1.01  (0.94, 1.08) | 1.28**  (1.11, 1.48) | 0.95**  (0.93, 0.98) | - |
| **Pig sex** |  |  |  |  |  |  |
| Female | Ref. | Ref. | Ref. | - | Ref. |  |
| Male | 0.80  (0.52, 1.23) | 0.94  (0.54, 1.66) | 1.03  (0.37, 2.93) | - | ***Village A:*** 5.10**  (1.72, 15.1)  ***Village B:*** 1.21  (0.80, 1.81)  ***Village C:*** 1.28  (0.57, 2.92) |  |
| **Season** |  |  |  |  |  |  |
| Rainy | Ref. | Ref. | Ref. | - | - | - |
| Dry | 0.86*  (0.72, 1.02) | 0.69  (0.39, 1.21) | 1.10  (0.39, 3.11) | - | - | - |
| **Herd size (per pig)** | 0.97  (0.93, 1.01) | 0.97  (0.91, 1.03) | 1.01  (0.91, 1.11) | - | - | - |
| **Pig age (per month)** | 0.99  (0.95, 1.02) | 0.98  (0.93, 1.03) | 1.01  (0.91, 1.12) | - | - | - |
| **Roaming land-type (per 1% increase by type)^c^** |  |  |  |  |  |  |
| Peri-domestic | 0.18**  (0.07, 0.43) | 0.17**  (0.05, 0.56) | 0.29  (0.04, 2.24) | - | - | - |
| Vegetation | 1.11  (0.41, 3.02) | 2.13**  (0.58, 7.81) | 1.62  (0.19, 13.8) | - | - | - |
| Roads/paths | 16.9**  (4.52, 63.1) | 5.74  (0.83, 39.7) | 2.34  0.10, 53.7) | 3.41**  (1.79, 6.49) | - | - |

p-value: **<0.01, *<0.05

^a^Significant statistical interactions (village*season)

^b^Number of households within 100m radius, linear spline at 25 households/100m

^c^Farming land-type not included due to insufficient roaming
